# From the Reclaimed Water Treatment Plant to Irrigation in Intensive Agriculture Farms: Assessment of the Fate of Antibiotics, Antibiotic Resistance Bacteria and Genes, and Microbial Pathogens at Real Scale

**DOI:** 10.1021/acs.est.5c02823

**Published:** 2025-09-09

**Authors:** Flor X. Cadena-Aponte, Patricia Plaza-Bolaños, Ana Agüera, Samira Nahim-Granados, Ilaria Berruti, María Jesús Abeledo-Lameiro, Maria Inmaculada Polo-López

**Affiliations:** † Solar Energy Research Centre (CIESOL), Joint Centre of the University of Almería-CIEMAT, Carretera de Sacramento s/n, Almería 04120, Spain; ‡ Department of Chemistry and Physics, University of Almería, Carretera de Sacramento s/n, Almería 04120, Spain; § Plataforma Solar de Almería-CIEMAT, Carretera de Senés Km 4, Tabernas 04200, Almería, Spain

**Keywords:** antibiotics, antibiotic-resistant genes, bacterial pathogens, chlorination, reclaimed water

## Abstract

This work aims to investigate the occurrence of 31 antibiotics (ABs), 2 bacteria (*Escherichia coli* and *Pseudomonas* spp.) and their counterpart antibiotic-resistant bacteria (carbapenem and cephalosporin families), and several antibiotic-resistant genes (ARGs) throughout a full distribution system of reclaimed water (RW) in a real-scale scenario. The RW was analyzed (i) before and after the tertiary treatment (sand filtration and chlorination), (ii) during the storage period in secondary ponds before its use in irrigation, and (iii) directly in the droppers installed in four plastic-based greenhouses over 9 months. The results obtained in RW showed a bacterial concentration below the minimum required to reach class A (<10 CFU/100 mL, Regulation EU 2020/741), a reduction of the initial AB concentration (up to 13 ABs, total 4847 ± 1413 ng/L) of 58%, and no significant reduction of ARGs (Log units/100 mL: 16S rRNA (9.99 ± 0.80) > *intI1* (8.80 ± 0.95) > *bla_CTX-M32_* (7.53 ± 0.63) > *sul1* (7.08 ± 1.05) > *bla*_*TEM*_ (6.81 ± 1.05) > *qnrS* (5.72 ± 0.82)). The storage of RW was a hotspot only for bacteria; an increase in all concentrations was observed in both main and secondary reservoirs, demonstrating that direct RW reuse is the most beneficial option to avoid significant bacterial regrowth. In all greenhouse droppers’ systems, a significantly higher concentration of all bacteria was generally detected than in secondary reservoirs, demonstrating that this is another hotspot independent of whether the RW is used directly or not. Therefore, the RW storage and distribution may negatively affect the microbial water quality, while ABs and ARGs are detected along the entire scheme of urban wastewater reclamation and reuse, reaching the greenhouse environment (including soil and plants).

## Graphical abstract

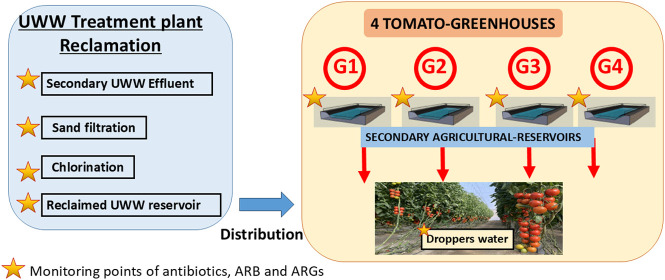

## 1 Introduction

The reuse of treated urban wastewater (UWW) for agricultural irrigation, especially in water-stressed regions, has gained attention in the last decades as a predictable and climate-independent source of water, providing nutrients that plants can directly assimilate, while lowering the pressure on water bodies and preserving them.^1–3^

However, it has been widely demonstrated that despite the fact that secondary treatment in urban wastewater treatment plants (UWWTPs) significantly reduce organic matter and enhance the water quality, secondary effluents still contain significant concentrations of pathogens and other targets of emerging concern, such as organic microcontaminants (OMCs) or antibiotic-resistant bacteria and genes (ARB and ARGs).^4^ Therefore, additional tertiary treatments are needed to reach the minimum requirements and safety standards, which are nowadays mandatory, to ensure environmental and human health protection.

In this respect, the European Union (EU) Regulation 2020/741 on water reuse establishes the regulatory framework for this activity, describing the use of treated water for agriculture and classifying different water quality classes (A–D) and a variety of end-uses to irrigate crops.^5^ For regulatory compliance, *Escherichia coli* is the used indicator of fecal contamination for assessing the microbial quality of UWWTP effluents.

The tertiary treatments commonly implemented at full scale in UWWTPs are based on processes, such as sand filtration in combination with chlorination, ozonation, and ultraviolet-C radiation. It is well-known that all of these treatments are effective for reducing the microbial concentration below the limits established in the wastewater guidelines and regulations.^6,7^ However, it has been demonstrated that the reclaimed urban wastewater (RW) obtained by these tertiary treatments constitutes a significant additional pathway for the introduction of OMCs, including antibiotics (ABs), in the agricultural environment,^8,9^ due to their low or limited removal rates in UWWTPs.^8^ The continuous discharge of OMCs into the environment poses potential long-term ecotoxicological effects on aquatic and terrestrial organisms and public health risks.^10^ Moreover, understanding the interactions between soil, water, plants (water–soil–plant nexus), and humans becomes a key aspect for future sustainability. The uptake and bioaccumulation of OMCs in the edible parts of final food products, their sorption and retention in soil, and their sequential entry into the human food chain could alter food safety, affecting human health.^9^

UWWTPs have also been recognized as potential hotspots for promoting the selection of ARB and ARGs due to the ideal environment with the simultaneous presence of high bacterial density input, nutrients, and ABs that are poorly removed with conventional treatments.^8,11^ Consequently, the use of RW for crop irrigation may result in the continuous exposure of the agricultural environment to ABs (and other OMCs), ARB, and ARGs. The development, occurrence, and dissemination of antibiotic resistance (AR) are current issues attracting great attention from the general public. In this sense, the AR phenomenon has been recognized as a global threat to health by the World Health Organization.^12^

As a result of the need for taking action to address both issues, the original Directive 91/271/EEC concerning UWW treatment has been revised, and the new Directive EU 2024/3019 ^13^ introduced the concept of a quaternary treatment to ensure the removal of a large spectrum of OMCs from UWW. A minimum degradation of 80% (in relation to the load of the influent) of at least six substances included in the Directive has been established. Moreover, regarding antimicrobial resistance, due to UWW is recognized as a major source of ARB and ARGs,^8^ the importance of tackling this issue has been outlined. A minimum frequency of sampling and a harmonized methodology for measuring antimicrobial resistance in UWW will be established and shall be adopted by 2 July 2026 (Directive EU 2024/3019).^13^ As regards disinfection byproducts (DBPs), they have not been regulated yet. Nevertheless, the (EU) Regulation 2020/741 draws attention to DBPs by including this category of contaminants among those that must be considered for risk management plan.^5^

According to Directive 2024/3019, the implementation of effective tertiary and quaternary water treatments will be mandatory (for facilities with ≥150,000 p.e.), being crucial to control not only the regulated chemical and microbiological contaminants, but also ABs, ARB, and ARGs.^13^ Different processes have been recently studied for their removal, including chlorination and ozonation (as typical tertiary treatments) and other innovative advanced oxidation processes (AOPs), such as ozonation/H_2_O_2_, homogeneous photocatalysis (photo-Fenton based on the use of iron (Fe^2+^ or Fe^3+^) and H_2_O_2_), and ultraviolet-C (UV-C) or solar-based processes with the addition of oxidants (including H_2_O_2_, peracetic acid, chlorine, peroxymonosulfate, and persulfate), among others.^14,15^ Nevertheless, most of these AOPs are under investigation and not implemented at full scale and/or applied as an actual source of treated water for irrigation in agriculture. Conventional chlorination is still the most implemented treatment for wastewater reclamation and reuse in UWWTPs,^15^ despite the well-known generation of DBPs (e.g., trihalomethanes and haloacetic acids).^16^

On the other hand, in the context of full-scale RW distribution and reuse for irrigation, the long-term maintenance of the microbial water quality until the point of use is currently considered an additional challenge. The unique physicochemical characteristics of RW, including high load of nutrients, fast decay of residual disinfectant, stagnation, and high distribution system retention times, eventually promote the recontamination of the treated water due to pathogens’ regrowth or postcontamination.^8,17^ In addition, more recently, the capability to promote antimicrobial resistance in RW has been reported, as stated by La Rosa.^11^ Nevertheless, full-scale RW distribution, few studies are found in the literature covering this matter at full-scale RW distribution. Fahrenfeld explored the occurrence of several ARGs, *Legionella pneumophila*, *E. coli*, and *Pseudomonas aeruginosa*at the point of entry and the point of use of three RW distribution systems in the United States (using filtration and chlorination or UV-C radiation as a tertiary treatment), demonstrating the potential for ARGs and waterborne bacterial pathogens to regrow within the RW distribution pipes.^6^ Truchado monitored the presence of indicator microorganisms (*E. coli*and spores of*Clostridium perfringens*) as well as pathogenic bacteria (Shiga toxin-producing*E. coli*,*E. coli* O157:H7, and *Salmonella* spp.) in different sampling points of three different RW systems (including influent and effluent of the UWWTPs, water reservoirs located at the distribution system, and the end-user point at the irrigation system). The RW was used to irrigate lettuce and baby spinach grown in open-to-the-air real crops.^7^ According to the study, the current technologies implemented in the UWWTPs (chlorination and UV-C ) provided RW with microbiological quality within established thresholds (average *E. coli* levels of all the samples from the UWWTP effluents of 0.73 ± 1.20 Log CFU/100 mL), but increasing levels of *E. coli* were found in the distribution/storage systems (average of 1.2 ± 0.9 Log CFU/100 mL in UWWTP reservoirs).^7^

The factors influencing contaminant translocation in the water–soil–plant nexus are not well-known because most of the studies in the literature are carried out at pilot scale and not always correlating with field conditions. In this sense, the wide variety of agricultural conditions (different crops, irrigation practices, reclamation treatments, soil characteristics, weather conditions, etc.) makes difficult to predict the behavior of contaminants in real-scale scenarios.^18^

The number of papers published related to OMCs monitoring in water reuse applications has increased significantly in the last years, especially from 2018 to nowadays, attributed to the increased global awareness of water scarcity and the increasing reuse of RW for agricultural irrigation. Nevertheless, as stated by Specker in a recent review, the number of studies focusing on its analysis at wastewater reclamation and reuse and at full-scale application is still relatively low. Only 48 papers were found by these authors in the period 2003–2023.^19^ In this line, the study by Martínez-Piernas can be highligthed as pioneer reporting the occurrence of OMCs (including 11 ABs) in a real RW treatment and reuse scenario including intensive tomato crop cultivation through the application of a wide-scope analytical approach based on a multianalyte target analysis (60 OMCs) and a suspect screening (>1300 OMCs), detecting 8 compounds in the final product.^20^ Recently (period 2024–2025), only a few studies have been found in the literature covering this topic,^21–24^ reinforcing the need for more data on real- and full-scale scenarios.

In this regard, a global analysis of the presence and fate of emerging concern targets (including bacteria, ARB, ARGs, and ABs) in different points of the water reclamation and reuse system in a real agricultural environment has not been conducted yet. This implies the need for a comprehensive assessment from the UWWTP to intensive agriculture irrigation sites, also paying attention to the potential regrowth along the water distribution system. Therefore, the objective of this work was the study of the occurrence and behavior of a set of bacteria, ABs, ARB, and ARGs, in a real intensive agricultural system devoted to the production of tomatoes in plastic-based greenhouses. The monitoring was performed in a reclamation urban wastewater treatment plant (RUWWTP, Almeria, Spain) and included the analysis of the target contaminants in selected sampling points throughout the RUWWTP system. Namely, UWW before and after the chlorination treatment and at four end-user greenhouses (including the secondary reservoirs placed on the farms and the droppers of the irrigation system in each greenhouse next to the tomato plants). The following parameters were analyzed: (i) physicochemical characterization; (ii) 31 ABs; (iii) two bacterial strains, *E. coli* (due to its status as a microbial indicator in water regulations and guidelines) and *Pseudomonas* spp. (due to its wide distribution in the environment and its recognition as an emerging opportunistic pathogen of clinical relevance),^25^ along with their AR counterparts (AR to carbapenem and cephalosporin families); and (iv) several ARGs, including *sul1*, *qnrS*, *bla_TEM_*, *bla*_*CTX-M32*_ (encoding resistance to sulfonamides, quinolone, β-lactamase, and cephalosporins, respectively), as well as the 16S rRNA gene (housekeeping) and the class 1 integron integrase *intI1* (a gene with a function of horizontal gene transfer), all selected for their prevalence in wastewater.^26^ Finally, the distribution and relationship of all of the collected data have been comprehensively evaluated, which allowed the identification of critical points in the distribution scheme focused on the potential microbial regrowth, ARGs spreading, and AB accumulation and degradation in a real-scale scenario of UWW reclamation and reuse in intensive agriculture.

## 2 Materials and Methods

### 2.1 Monitoring Scheme Description

The water sample monitoring of this study was carried out for 9 months in Almeria (Southeast of Spain) and included the following sampling points, depicted in Figure S1:

(i) Secondary effluent (UWW) obtained from El Bobar UWWTP, with a capacity of 315,000 p.e. It consists of a conventional plant with a pretreatment step, a primary treatment, and a secondary treatment based on the activated sludge process. The sampling was done at the entrance of the next step in the RUWWTP. A total of 10 samples were collected and analyzed from September to November 2020 (4 samples per month).
(ii) RW from the RUWWTP: it consists of sequential filtration (sand and anthracite filters) and chlorination (NaClO) to meet the requirements of water quality according to the Spanish regulation for water reuse,^27^ the in-force regulation in Spain at the time of this study. The produced RW is then stored in a main reservoir (120,000 m^3^) and distributed to the greenhouses on demand. Ten samples were taken in September–November 2020: (i) after the sand filtration step (RW-F), (ii) after chlorination (RW-Ch), and (iii) in the main reservoir (RW-R). Besides, the sampling of RW-R was expanded to cover the same period of analysis as the RW used in the greenhouses. Therefore, 23 additional samples were collected from November 2020 to April 2021 (2–3 samples/month).
(iii) RW at the agricultural end-user: samples were taken from four anonymous plastic-based greenhouses (G1, G2, G3, and G4) devoted to tomato cultivation and connected to the distribution systems of the RUWWTP. Each greenhouse was managed independently by each farmer. Water samples were collected from November 2020 to April 2021 (around 2 samples/month) from (i) the secondary reservoir (R1, 10 samples; R2 ,13 samples; R3, 13 samples and R4, 10 samples) and (ii) water outlet from the drip irrigation system (droppers, D) placed on the soil next to the tomato plant (D1, 11 samples; D2, 14 samples; D3, 14 samples and D4, 11 samples).

Water use practices in each greenhouse were different as summarized below: (i) G1 (cultivation area, 6000 m^2^): direct use of RW without storage; a reservoir open to the atmosphere and filled with well water was available for water contingencies (R1, 950 m^3^); (ii) G2 (cultivation area, 3000 m^2^): RW storage in a reservoir open to the atmosphere (R2, 200 m^3^), with an average storage time of 1 month; (iii) G3 (cultivation area, 2200 m^2^): RW storage in a reservoir covered with duckweed (R3, 200 m^3^), with an average storage time of 1 month; (iv) G4 (cultivation area, 5000 m^2^): mix of groundwater and RW (1:3 *v/v*) and storage in a reservoir open to the atmosphere (R4, 400 m^3^), with an average storage time of 2 weeks.

All water samples were characterized according to common water parameters. Turbidity, pH, and conductivity were analyzed by a turbidimeter (2100AN, Hach, Loveland, CO), a pH-meter (LAQUAact-110-K, Horiba Advanced Techno Co., Ltd., Osaka, Japan), and a conductimeter (GLP31, Crison, Barcelona, Spain), respectively. Dissolved organic carbon (DOC) and bicarbonate ions (measured as inorganic carbon) were analyzed using a total organic carbon analyzer (TOC-L-CSN, Shimadzu, Kyoto, Japan). Water ionic content was determined by ion chromatography (850 with the 872 extension module, Metrohm, Herisau, Switzerland). Free chlorine was quantified according to a Hach spectrophotometric procedure (Method 10069, equivalent to US-EPA method 330.5 for wastewater) based on a colorimetric DPD (*N*,*N*-diethyl-*p*-phenylenediamine) oxidation and using the associated reagent and instrument (DR 300 Chlorine PocketColorimeter and DPD Free Chlorine Reagent Powder Pillows Hach PN 1407099, Hach).

### 2.2 Analysis of Antibiotics (ABs) by LC-MS/MS

The determination of the selected ABs in the water samples was carried out by liquid chromatography coupled to a quadrupole-linear-ion-trap mass spectrometry (LC-QqLIT-MS/MS) using the direct injection (DI) technique. An Exion AC liquid chromatograph (Sciex, Foster City, CA) and a 5500 QTRAP (Sciex) mass spectrometer were used. LC-MS degree solvents were supplied by Honeywell (Hannover, Germany). High-purity AB standards (>95%) were purchased from Sigma-Aldrich (Darmstadt, Germany), Dr. Ehrenstorfer GmbH (Augsburg, Germany), Fluka (Steinheim, Germany), HPC (Cunnersdorf, Germany), and TRC Canada (Toronto, Canada). ^13^C-Caffeine was used as an internal standard (injection standard). Matrix-matched calibration was used for AB quantification (*R* ^2^ always ≥0.9900). Precision was evaluated in terms of relative standard deviation (RSD, intraday) in spiked samples (reclaimed water, 50 ng/L) and was always ≤25%. Full description of the LC-MS/MS method and AB quantification can be found in Content S1 and Table S1.

### 2.3 Quantification of Bacteria and Antibiotic-Resistant Bacteria (ARB)

Bacterial analysis was performed by the standard plate counting and membrane filtration method using specific and selective agar, using Pseudomonas Chromogenic Agar (Condalab, Madrid, Spain) and ChromoCult Coliform Agar (Merck KGaA, Darmstadt, Germany) as selective media to quantify *Pseudomonas* spp. and *E. coli*, respectively. Sample volumes from 50 to 500 μL were spread on the selective media (limit of detection, LOD: 2 CFU/mL). For lower bacterial load, 100 mL of water sample (LOD: 1 CFU/100 mL) was analyzed by the membrane filtration method using the same agar media, cellulose nitrate microfilters (0.45 μm, Sartorius Stedim, Guxhagen, Germany), and a microfiltration system (Millipore, Bedford, MA). Plate colonies were counted after incubation for 24 and 48 h at 37 and 35 °C for *E. coli* and *Pseudomonas* spp., respectively. ARB was quantified by a similar procedure with an incubation time of 72 h and using the selective agar media supplemented with antibiotics according to the EUCAST database (January 2020): only carbapenem antibiotics (8 mg/L of meropenem and 4 mg/L of imipenem (both from Sigma-Aldrich)) for AR-*Pseudomonas* and with cephalosporins and carbapenem antibiotics (16 mg/L of cephalexin, 24 mg/L of cefotaxime, 8 mg/L of meropenem, and 4 mg/L of imipenem (all from Sigma-Aldrich)) for AR-*E. coli*.

### 2.4 Antibiotic-Resistant Genes (ARGs)

A 100 mL sample aliquot was filtered in duplicate through a 0.2-μm polycarbonate membrane (Chmlab Group S.L., Barcelona, Spain), and the total DNA was extracted using the DNeasy PowerWater kit (Qiagen Sciences Inc., Germantown, MD), according to the manufacturer’s instructions. The total DNA obtained was then measured using a NanoDrop Lite spectrophotometer (Thermo Fisher Scientific, Waltham, MA) and stored at −20 °C for further analysis.

The PCR amplification was performed using PowerUpSYBR Green Master Mix 2 (Applied Biosystems, Thermo Fisher Scientific, Vinius, Lithuania) in a quantitative real-time PCR (qPCR) (7500 Fast Real-Time PCR Systems, Applied Biosystems, Thermo Fisher Scientific) using the working conditions and quantification procedure described in previous works.^28,29^ In addition, more details can be found in Content S2 and Table S2.

### 2.5 Statistical Analysis

All data in figures and tables are shown as the averaged value with the corresponding error as the standard deviation. The one-way repeated measures ANOVA statistical analysis of the data was accomplished using OriginPro (2023, version 10.0.0.154, OriginLab Corporation, Northampton, MA). Significant differences between replicates were considered at *p*-values <0.05 (ANOVA). In addition, the ANOVA analysis was complemented by the Bonferroni test, which allows the simultaneous identification of significant differences of the data means analyzed, which is estimated at *p*-values <0.05. The median and interquartile range (IQR) of the data were also calculated using OriginPro. The box plots of the graphs show the median with the 25^th^ and 75^th^ percentile values of the data reduction.

## 3 Results and Discussion

### 3.1 Physicochemical Analysis

#### 3.1.1 Water Reclamation Treatment Plant

Knowing the physicochemical characteristics of a water source used for agricultural irrigation is important from an environmental and agronomic point of view. Thus, the analysis of the UWW along the different stages of the reclamation plant was performed (Table S3) to evaluate the effect of the treatment on its properties. The sand filtration step did not generate any significant change in the physicochemical parameters of the wastewater. No statistically significant differences (*p*-value >0.05) were observed during the studied period for the analyzed parameters along the treatment steps, except for NO_2_^–^ (1.8 ± 1.0 and 1.0 ± 0.6 mg/L for UWW and RW-F, respectively). The impact on the turbidity, expected to be noticeable after a filtration process, showed only a decrease of 1.8 NTU from 7.4 ± 1.8 to 5.6 ± 2.1 NTU. The low impact on this parameter could be attributed to the low initial turbidity value.

In the subsequent chlorination stage, statistically significant variations were only observed for turbidity (4.3 ± 1.2 NTU), which was reduced to 3.0 NTU with respect to UWW (7.4 ± 1.8 NTU), and for the ammonia content (66.5 ± 13.8 mg/L), which was significantly reduced by 3 mg/L compared to the initial value in UWW (NH_4_^+^, 69.2 ± 16.4 mg/L). Although ammonia reduction was expected due to chloramine generation, a slight turbidity decrease observed after chlorination (from 5.6 ± 2.1 to 4.3 ± 1.2 NTU) was also notable. As the capability of chlorination to oxidize the UWW organic content has been previously reported,^30^ an indirect effect in the turbidity value derived from chlorine oxidation of some particulate organic matter fractions, as well as a slight settling of particles during the treatment, cannot be discarded.

The highest statistically significant variations were, however, observed in the main reservoir (RW-R) for HCO_3_^–^ 537.9 ± 83.6 mg/L, NO_2_^–^ 4.8 ± 7.3 mg/L, and NH_4_^+^ 74.9 ± 16.4 mg/L, showing higher values in all cases in comparison with UWW and RW-Ch. A slightly higher average value for DOC (19.2 ± 4.4 mg/L) was also observed. This increase can be explained by the fact that the reservoir is open to the atmosphere, including the possibility of algae formation due to eutrophication, contamination by external factors such as animals or wind transport, and particle deposition.^31^

Regarding agronomic characteristics of RW, the presence of macronutrients (nitrogen, phosphorus, and potassium) and DOC content is beneficial for both the farmers (by reducing the need for commercial fertilizer^2^) and the crops, as pH (7.6 ± 0.2), turbidity (6.4 ± 3.0 NTU), and HCO_3_^–^ (537.9 ± 83.6 mg/L, i.e., ca. 8.8 mEq/L) values agreed with the recommended water quality for irrigation (pH of 6.5–8.5, <30 NTU and <10 mEq/L of HCO_3_^–^).^32^ The conductivity value (3.0 ± 0.3 mS/cm) was in the upper limit of the recommendations. However, the RW analyzed is used for tomato cultivation, a crop that is moderately sensitive to salinity and for which the conductivity value is within the recommended quality thresholds and yield (even a higher value can improve the compositional and sensory properties of this fruit).^33,34^ Apart from salinity, the most common problems associated with irrigation water are related to certain ions (SO_4_^2–^, Cl^–^, Na^+^, Mg^2+^, and Ca^2+^) and the concentration ratio between some of them, which can affect soil properties. High concentrations of SO_4_^2–^, Cl^–^, and Na^+^ (>20, 30, and 40 mEq/L, respectively) can be toxic to crops and can imbalance the availability of other essential nutrients such as phosphorus or potassium. The concentration of these ions in the RW analyzed (<20 mEq/L) was below these recommended values.^32^ To study the quality of irrigation water in relation to the suitability of the soil for crop production, two indices are generally used: (i) the residual sodium carbonate (RSC), which can assess the alkalinity risk for the soil according to the concentrations of HCO_3_^–^, Mg^2+^, and Ca^2+^ and (ii) the sodium adsorption ratio (SAR), which, together with conductivity, allows the evaluation of the permeability/infiltration rate of the water into the soil according to a relationship between Na^+^, Mg^2+^, and Ca^2+^ concentrations. The RSC (ca. 0 mEq/L) and SAR values (ca. 6 mEq/L and ca. 3 mS/cm) for the RW of this study were within the recommended limit values for both indices: <0.5 mEq/L for the RSC and conductivity >1.9 mS/cm for an SAR value of 6 mEq/L.^32^

#### 3.1.2 Greenhouses

As stated above, the physicochemical characteristics of the RW are not significantly affected by the reclamation treatment (filtration and chlorination) or storage in the reclamation plant. However, during the distribution stage, the different water management practices applied in each greenhouse can affect the quality and characteristics of the water that the tomato plants finally receive. Therefore, the physicochemical characteristics of RW were monitored in each selected greenhouse, including the secondary reservoirs R1 (with 100% groundwater), R2 (100% RW), R3 (100% RW), and R4 (75% groundwater-25% RW) and the drip water (D1, D2, D3, and D4, all using RW) (Figure 1 and Table S4 including statistical analysis). The cationic and anionic contents are also summarized in Table S5.

**Figure 1:**
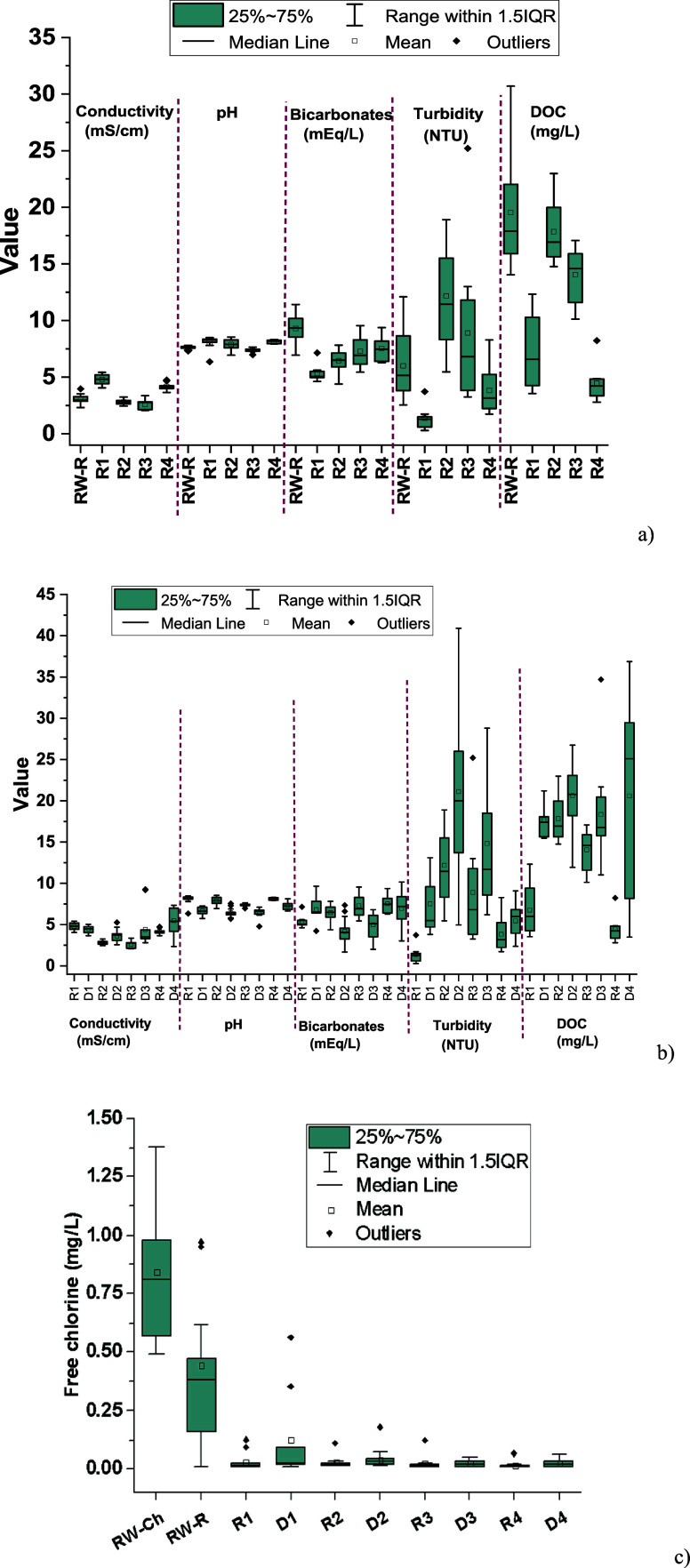
Comparison of the physicochemical parameters of the four greenhouses: (a) RW-R versus secondary reservoirs (R), (b) secondary reservoirs (R) versus dropper water (D), and (c) free chlorine concentration.

Figure 1a shows the values of the main physicochemical parameters of the secondary reservoirs compared to RW-R. As expected, R1 and R4 showed the greatest differences, attributed to (i) the specific characteristics of groundwater (high salinity content, low turbidity (<2 NTU) and almost absence of DOC (<1–5 mg/L) (Table S4)) and (ii) their composition, 100 and 75% of groundwater, respectively. On the other hand, no quality changes were observed in the case of R2 and R3 (100% RW) due to the storage time, with a few exceptions. Turbidity slightly increased in R2 (12.2 NTU) and R3 (8.9 NTU) compared to that in RW-R (6.0 NTU). The behavior in R2 can be attributed to the accumulation of nutrient-rich RW in an open-to-the-atmosphere reservoir, which increases the possibility of algae formation and soiling due to external factors. In the case of R3, the presence of a duckweed cover contributes to protecting the water from external factors, favoring a lower impact on turbidity. Furthermore, the bioremediation capacity of these plants may explain the lower DOC observed.^35^ Comparing the physicochemical characteristics of the RW collected in the drip irrigation system with those corresponding to the reservoir (Figure 1b), a significant decrease in both HCO_3_^–^ and pH values was observed in all greenhouses. This drop-off can be explained by the common agricultural practice of adding phosphoric acid to the irrigation water to provide phosphorus and reduce water alkalinity, thus avoiding nutrient imbalances and the precipitation of carbonate salts.^32^

DOC and turbidity also increased in all dripper water samples, except D1. These increases can be explained by clogging due to the enhanced biological growth rates observed in the irrigation lines and drippers. Such growth is a consequence of the high nutrient levels, organic matter, and endogenous bacterial content of RW, which causes the rapid formation of algae and biofilms.^32,36^ The preservation of the physicochemical quality observed in D1, where the irrigation was carried out directly from the reclamation plant without any storage, can be related to the eventual presence of residual free chlorine (0.3–0.5 mg/L) in this RW (Figure 1c), unlike the other greenhouses, where free chlorine was depleted during the RW storage. In fact, chlorination at a low concentration (0.4–2.0 mg/L) is the most effective and commonly applied method to control the growth of microorganisms and prevent bioclogging in drip irrigation systems.^37^

The increase in conductivity observed in all of the dropper water samples in comparison with the secondary reservoir is due to the fertilizer addition and the presence of saline groundwater in the case of D1 and D4 (Figure 1b). It is important to note that fertigation is a widespread practice in intensive greenhouse agriculture to increase the bioavailability of both macro- (N,K,P) and micronutrients (Ca^2+^) to the crop. This is done by adding different salts such as KNO_3_, NH_4_NO_3_, Ca­(NO_3_)_2_, NH_4_H_2_PO_4_, and the previously mentioned H_3_PO_4_, as can be observed by the increase in the concentrations of NO_3_^–^, PO_4_^3^, NH_4_^+^, K^+^, and Ca^2+^ in the drip water samples analyzed (Table S5).^38^

### 3.2 Analysis of Antibiotics

#### 3.2.1 Water Reclamation Treatment Plant

The presence of 31 target ABs from different classes, including aminopenicillins (1), cephalosporins (2), (fluoro)­quinolones (9), lincosamides (2), macrolides (5), nitroimidazoles (1), sulfonamides (7), and tetracyclines (4), was investigated. Up to 13 ABs were detected at the inlet of the tertiary treatment plant, representing a total ABs load of 4847 ± 1413 ng/L, as shown in Table 1. Most of the compounds were present in all samples (*n* = 10) throughout the sampling period, except for clindamycin, enrofloxacin, and tetracycline, which were detected only in October and November (60% detection frequency). Ciprofloxacin (2113 ± 862 ng/L), levofloxacin (expressed as the sum of the enantiomers levofloxacin and ofloxacin, not chromatographically resolved in the applied method; 1054 ± 344 ng/L), and azithromycin (711 ± 435 ng/L) reached the higher average concentrations, representing 80% of the total load. These results are in line with previous studies^39^ and with the pattern of antibiotics consumption in Spain, considering tetracyclines, macrolides/lincosamides, sulfonamides (β-lactams included in this study were not detected), although this pattern can vary depending on the age range (e.g., penicillins are mostly consumed among young population, from 0 to 14 years old).^40^

**Table 1:** Average Levels of ABs (in ng/L) Detected in the Sampling Points of the Tertiary Treatment Plant

	UWW				RW-F				RW-Ch				RW-R				
	ATL^a^	SD^b^	FD (%)^c^	min–max^d^	ATL	SD	FD (%)	min–max	ATL	SD	FD (%)	min–max	ATL	SD	FD (%)	min–max	removal (%)
**Σ antibiotics**	**4847**	**1413**	**-**	**3338–7591**	**4660**	**1800**	**-**	**1796–7807**	**2050**	**813**	**-**	**977–3536**	**1981**	**1018**	**-**	**695–3723**	**58**
Azithromycin	711	435	100	236–1520	695	437	100	146–1454	726	497	90	243–1580	778	497	800	154–1568	0
Ciprofloxacin	2113	862	100	1112–3704	2023	1038	100	725–3895	n.d.	-	-	-	n.d.	-	-	-	100
Clarithromycin	22	10	100	10–35	19	10	100	10–35	24	8	80	13–33	20	7	90	10–27	0
Clindamycin	23	8	60	11–36	30	15	60	16–52	n.d.	-	-	-	n.d.	-	-	-	100
Enrofloxacin	30	16	60	12–50	31	19	60	13–60	25	16	60	11–48	27	19	60	10–58	14
Erythromycin	70	21	100	43–105	63	13	100	47–78	60	14	100	31–75	62	23	100	30–115	14
Levofloxacin^e^	1054	344	100	734–1619	1024	426	100	400–1650	1000	335	100	552–1462	954	470	90	248–1579	5
Lincomycin	141	68	100	32–233	131	65	100	31–243	n.d	-	-	-	n.d.	-	-	-	100
Metronidazole	79	23	100	45–115	60	23	100	25–101	63	24	100	37–106	52	15	100	25–70	19
Sulfamethoxazole	376	137	100	184–545	366	138	100	202–587	166	111	100	44–335	218	146	100	69–468	56
Sulfapyridine	90	51	100	32–173	95	50	100	35–184	36	16	80	11–64	64	49	100	10–134	60
Tetracycline	100	31	60	56–143	104	34	60	63–149	n.d	-	-	-	n.d.	-	-	-	100
Trimethoprim	101	58	100	27–161	86	49	100	19–152	47	39	100	17–125	70	51	100	17–181	53

a ATL: average total load (*n* = 10).

b SD: standard deviation.

c FD: frequency of detection.

d Concentration range showing the minimum and maximum concentrations.

e Sum of levofloxacin and the enantiomer ofloxacin. n.d.: not detected.

Regarding the behavior of the ABs identified during the tertiary treatment line, no significant difference (*p*-value 1) on the total AB load was observed after the filtration step (RW-F in Table 1), while statistical differences between RW-F and RW-Ch were obtained (-value 0.0005), with an average removal rate of 58% achieved after the chlorination treatment. However, this removal rate varied considerably depending on the compound. Thus, the total removal of clindamycin, lincomycin, and tetracycline, especially of ciprofloxacin, present at the highest concentration in the secondary effluent, was noteworthy. This reduction may be due to the formation of transformation products (also known as disinfection byproducts (DBPs) or chlorinated-DBPs when containing chlorine) by the oxidant properties of chlorine as it was already reported for ciprofloxacin, including the opening of the piperazine ring in the original molecule and the addition of chlorine in some of the generated compounds.^41,42^ In contrast, azithromycin, levofloxacin, and other less abundant ABs (clarithromycin, erythromycin, and enrofloxacin) remained almost unchanged (<14% removal) or exhibited low/moderate degradation rates, ranging from 19% (metronidazole) to 60% (sulfapyridine). The absence of degradation after the chlorination process observed for azithromycin is not in agreement with the results reported for laboratory chlorination experiments, which might suggest the influence of other unknown factors on degradation.^41^ Nevertheless, regarding the total load of ABs, no significant difference (-value 1) was observed between RW-Ch and RW-R, standing out in its presence and stability along the storage time.

The obtained results confirm the inefficiency of the chlorination treatment for the total elimination of ABs, pointing to azithromycin, levofloxacin, and sulfamethoxazole as the main ABs present in irrigation water. The poor AB removal efficiency shown by the chlorination process outlines the need for replacing this extended treatment with an alternative one with a higher removal capability. In this regard, several processes are being studied for OMC removal in UWW, such as ozonation, activated carbon (AC), membrane filtration, photochemical AOPs (lamp or sunlight-based), or electrochemical oxidation, among others.^43^ Among them, and discarding the processes that are still in a low-intermediate technology readiness level (TRL), the most consolidated and efficient alternative treatment until now is ozonation, which has shown high OMC removal efficiency (>80%) with low ozone doses (2–13 g O_3_/m^3^) and a moderate cost (ca. 0.1 €/m^3^).^44,45^

#### 3.2.2 Greenhouses

Concerning AB levels in the secondary reservoirs (Figure 2 and Table S6), significant differences compared with RW-R were observed, depending on the agricultural practices applied. For R1, a significant difference with RW-R was observed (*p*-value 0.0198), which can be explained by the fact that it is usually filled with groundwater, and only traces of azithromycin, levofloxacin, and trimethoprim were eventually detected, reaching a very small average load of 44 ng/L. This load increased to 88 ng/L in R4 when the groundwater was mixed with RW (25%) and showed a significant difference regarding RW-R (*p*-value <0.0001). Concentrations found in R2 and R3, using RW without mixing, were generally comparable and higher than the other reservoirs (1548 and 1809 ng/L, respectively), and no significant differences were observed among them and regarding RW-R as well. The lower concentration in the open reservoir (R2) suggests that photolysis phenomena can favor AB degradation, while a duckweed cover was used in R3 that may act as a light screen, limiting the photolysis effect and explaining the slight differences in AB concentrations. Besides, only for R3, significant differences were observed in comparison with R1 and R4 (*p*-values 0.0023 and 0.0027, respectively). Current data support that, in general, ABs can be affected by direct photolysis at different levels.^46,47^

**Figure 2:**
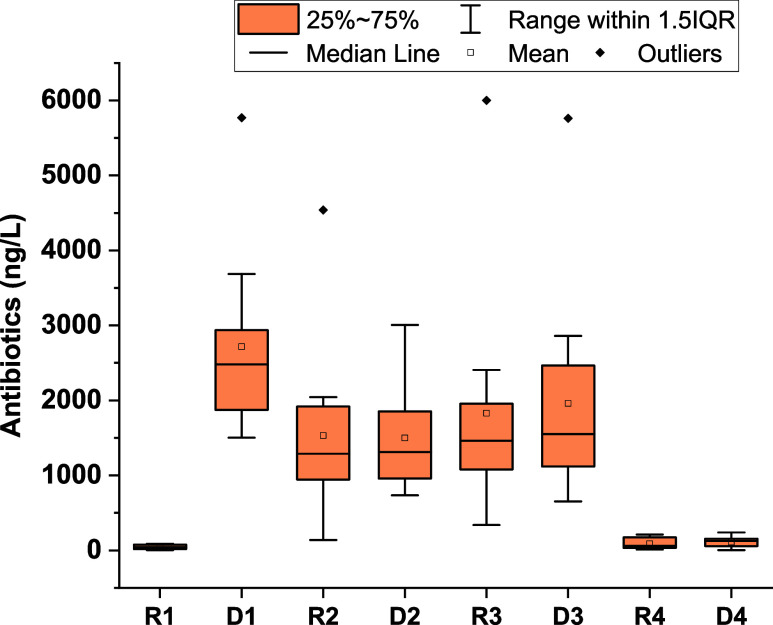
Average total levels of ABs detected in secondary reservoirs (R) and droppers (D).

No statistical differences on the AB levels were detected between the droppers and the corresponding secondary reservoirs (Figure 2 and Table S7) in greenhouses 2, 3, and 4 due to AB concentrations were similar, and therefore, this indicates that despite the farmers’ activities, ABs are not significantly accumulated in the dropper systems along the cultivation period. However, as expected, in the case of D1, which supplied RW directly from the treatment plant without prior storage, a significant difference was observed with R1 (*p*-value R1–D1 < 0.0001). Besides, in D1, the total AB load was considerably higher in comparison with D2, D3, and D4, confirming once again that storage contributes to reducing the AB concentration in irrigation water.

### 3.3 Microbiological Analysis

#### 3.3.1 Water Reclamation Treatment Plant

Levels of *E. coli*, *Pseudomonas* spp., and their AR counterparts in water samples at each sampling point through the water reuse system are shown in Figure 3. The median concentration of *E. coli* in the UWW was 5.5 Log CFU/100 mL (Figure 3a and Table S8). The water reclamation treatment, involving sand filtration and chlorination (NaClO, ca. 1 mg/L free chlorine), significantly reduced *E. coli* loads to 0.8 Log CFU/100 mL in RW-Ch. The overall ANOVA analysis revealed that the *E. coli* population showed significant differences (*p*-value 0.001), specifically attributed to the differences between RW-F and RW-Ch (*p*-value <0.05). No significant differences between UWW and RW-F and between RW-Ch and RW-R were observed (Table S8). 100% of the RW-Ch samples complied with the minimum quality requirements established for this indicator bacterium in the EU legislation (category C, *E. coli* ≤ 1000 CFU/100 mL, drip irrigation).^5^ Nevertheless, a slight increase in the microbiological load (1 Log CFU/100 mL), 6 out of 30 outliers, and 13% of samples with a concentration above the EU legislated threshold were detected in the main reservoir (RW-R). This is due to elevated storage times that were dependent on the farmers’ demand (ranging from 24 h to a week, depending on the crop demand), highlighting the deterioration in water quality during the storage and the control of the distribution system as a major challenge, as it has been previously reported.^7,48^

**Figure 3:**
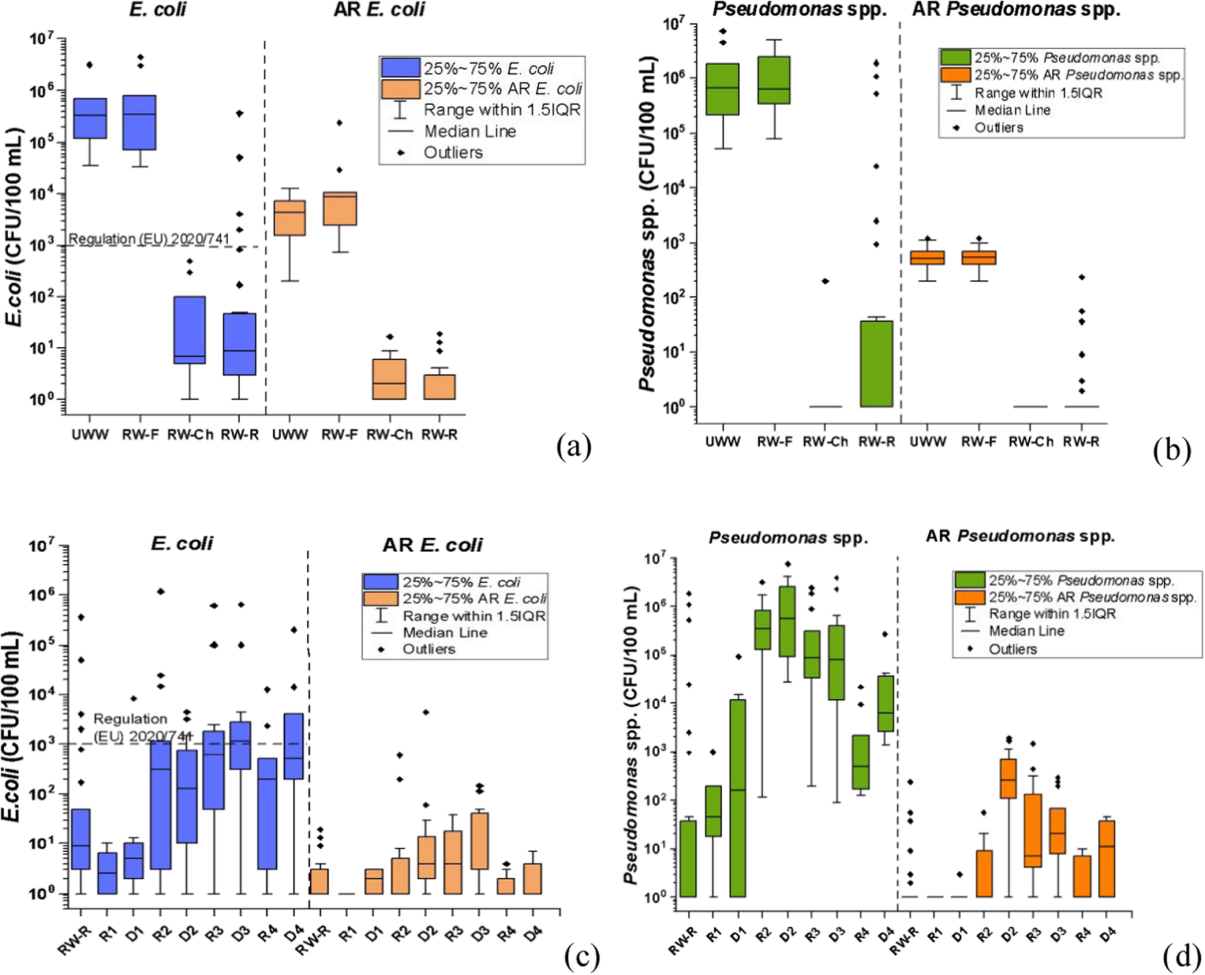
Box chart of median, outliers, and percentiles (25,75) of (a) *E. coli* and AR-*E. coli*, (b) *Pseudomonas* spp. and AR-*Pseudomonas* spp. concentrations at each sampling point in the reclamation plant; (c) *E. coli* and AR-*E. coli*; and (d) *Pseudomonas* spp. and AR-*Pseudomonas* spp. concentrations in RW-R, secondary reservoirs (R) and drip irrigation system (D) in the four greenhouses.

Effective inactivation of *Pseudomonas* spp. (with 6 logarithm reduction values (LRVs)) was achieved after the chlorination step, reaching a median value of 1 CFU/100 mL. Significant differences (*p*-value <0.001) were also observed between RW-F and RW-Ch (*p*-value <0.05), with no statistical differences between the other treatment steps. However, a large increase in its concentration (median value of 1.5 Log CFU/100 mL) was observed in the RW-R (Figure 3b). Unlike most indicator bacteria, some opportunistic pathogens, *Pseudomonas* spp. among them, regrow to a higher concentration in the distribution system, surviving within biofilms.^49,50^ Therefore, it should be essential to control the residual disinfectant, ensure efficient removal of organic matter content, and improve biological stability to prevent microbial regrowth in the RW distribution system, thus ensuring its safe use.^51^

Moreover, the RW distribution system may represent a potential source and spread of ARB and ARGs in the environment and possible transfer through the food chain has been identified as another important issue to be addressed.^52^ It has also been recognized as an additional requirement of particular concern in EU Regulation 2020/741, but minimum requirements have not been set yet.^5^ 100% of UWW samples were positive for AR-*E. coli* and AR-*Pseudomonas* spp. (Figure 3a,b), with a median count of 3.6 and 2.7 Log CFU/100 mL, respectively. It is noteworthy that, in relation with wild bacteria, the average percentage detected in UWW was 0.80 ± 0.50 and 0.19 ± 0.30 for AR-*E. coli* and AR-*Pseudomonas* spp. This highlights that less than 1% of the investigated bacteria exhibited specific resistance to cephalosporins and carbapenem antibiotics. In the subsequent treatment steps, both AR-bacterial concentrations decreased to ≤10 CFU/100 mL (stricter limit for quality class A, EU 2020/741)^5^ in RW-Ch and remained stable in RW-R (with only 10% of samples with higher values for both bacteria).

#### 3.3.2 Greenhouses: Secondary Reservoirs and Drip Irrigation System

Regarding the results of the reservoirs (Figure 3c,d), the microbial concentration detected in R1 was much lower compared to the others, which was expected as it was filled with groundwater (0.3 and 1.6 Log CFU/100 mL for *E. coli* and *Pseudomonas* spp., respectively). Nevertheless, similar results for all bacteria were obtained in the reservoirs of greenhouses (G2–4), where a secondary reservoir was used to store the RW (ca. 1 month). An increase in microbiological concentration was detected in water samples from R2–4 compared to RW-R, but the overall ANOVA analysis showed no statistical differences in the data means at the level of 0.05, with a few exceptions only in the case of *Pseudomonas* spp. (Table S8). Despite this, the median *E. coli* load (2.3–2.8 Log CFU/100 mL) complied with the water reuse regulation, with 31, 23, and 20% of the samples exceeding this threshold for R2, R3, and R4, respectively. *Pseudomonas* spp. exhibited the largest increased concentration with the following order: 2.7, 4.9, and 5.5 Log CFU/100 mL for R4, R3, and R2, respectively. Regarding AR-*E. coli* and AR*-Pseudomonas* spp., a similar tendency was observed in all reservoirs, but with significantly lower concentrations (<10 CFU/100 mL).

On the other hand, in G1, as the irrigation was performed directly from the distribution system, a similar average microbial concentration in the dropper system to that obtained in RW-R was detected (*E. coli* load of 0.7 Log CFU/100 mL and ≤ 10 CFU/100 mL of AR-*E. coli* and AR-*Pseudomonas* spp.), except for *Pseudomonas* spp., where an average of 1.6 Log CFU/100 mL was detected in comparison with RW-R.

The comparison between secondary reservoirs and dropper systems from G2-G4 shows a similar or higher microbial concentration in the droppers, highlighting an average of 0.3 Log CFU/100 mL higher for *E. coli* (in D3 and D4), 0.6 for AR-*E. coli* (in D2), 0.2 and 1.1 for *Pseudomonas* spp. (in D2 and D4, respectively), and 2.4, 0.5, and 1 for AR*-Pseudomonas* (in D2, D3, and D4, respectively). Chlorination has been reported in the literature as a potential promoter of the antimicrobial resistance of bacteria, particularly selectively in the case of *Pseudomona*s spp.,^53^ which may explain the higher prevalence of AR-*Pseudomonas* spp. than AR-*E. coli* detected in our study.

This result confirmed that the direct use of the RW could be a safer practice. Furthermore, the maintenance of RW quality during storage and distribution is a critical issue that should be carefully controlled to prevent bacterial regrowth. Water quality deterioration after distribution and storage could be mainly related to the presence of nutrients and high organic matter (Figure 1a), chlorine depletion, increased storage time in the distribution system, and poor microbial quality in storage facilities and distribution pipes, with biofilm accumulation and potential animal intrusion.^18,49,50^ On the other hand, the importance of analyzing the farmers’ pipelines and drip irrigation systems is also emphasized. In this regard, efforts should be made to promote a more comprehensive maintenance and cleaning program in greenhouses to enhance the effectiveness of RW reuse for agriculture. Besides, to prevent the accumulation of potentially harmful microbial populations, other microbial pathogens should also be considered apart from the commonly used *E. coli* indicator, particularly those capable of generating biofilms, such as *Pseudomonas* spp.^50^ In fact, in this study, a significantly high concentration of *Pseudomonas* spp. regarding *E. coli* in the four greenhouse dropper systems has been demonstrated, independently of the direct use (G1) or not (G2–4) of the RW.

### 3.4 ARG Analysis

#### 3.4.1 Water Reclamation Treatment Plant

Bacterial 16S rRNA gene and all ARGs detected in UWW followed the concentration ranking (based on average concentrations in Log units/100 mL): 16S rRNA (10.46 ± 0.26) > *intI1* (9.52 ± 0.60) > *bla_CTX-M32_* (8.08 ± 0.38) > *sul1* (7.91 ± 0.48) > *bla_TEM_* (7.35 ± 0.75) > *qnrS* (6.20 ± 1.01) (Figure 4). These results are consistent with those in the literature, where a wide range of concentrations for ARGs in the effluents of UWWTPs have been reported. Manaia reported that, from 100 full-scale UWWTPs analyzed around the world, concentrations ranged from 7 to 11 Log units/100 mL for bacterial 16S rRNA and concentrations of ARGs up to 9 Log units/100 mL, regardless of the type of treatment lines.^54^

**Figure 4:**
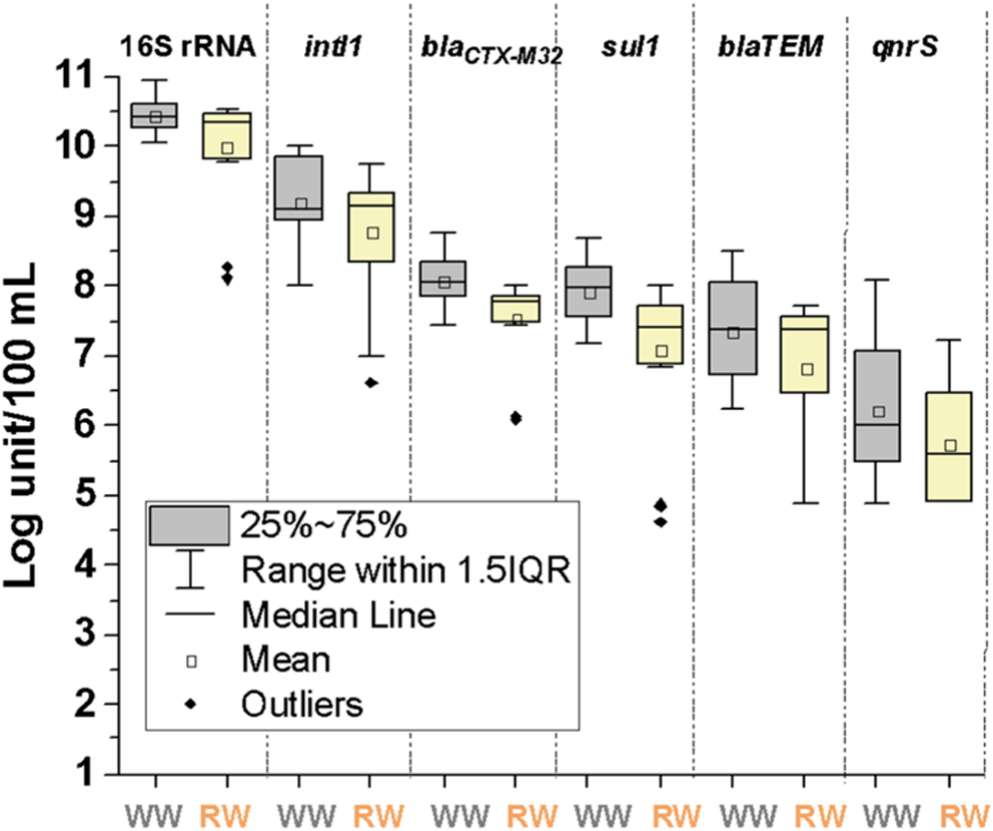
Quantification of ARGs in UWW and RW-R in Log units/100 mL.

After chlorination (RW-R), a similar order of detection was obtained: 16S rRNA (9.99 ± 0.80) > *intI1* (8.80 ± 0.95) > *bla_CTX-M32_* (7.53 ± 0.63) > *sul1* (7.08 ± 1.05) > *bla_TEM_* (6.81 ± 1.05) > *qnrS* (5.72 ± 0.82). These results agreed with other authors who reported similar gene concentrations in RW. Rocha reported 4 and 5 Log units copies/mL of *intI1* and *sul1* in a simulated RW distribution system.^55^ Keenum detected concentration ranges of 2–6 Log units/mL of *intI1* and *sul1* and 2–4 Log units/mL of *bla_CTX-M_* in RW.^56^

Although the concentration of all genes detected in RW-R was lower than in UWW, only a slight decrease was observed: 0.47, 0.72, 0.55, 0.83, 0.54, and 0.48 Log reduction values for 16S rRNA, *intI1*, *bla_CTX-M32_*, *sul1*, *bla_TEM_*, and *qnrS*, respectively, and statistical differences (*p*-values) between UWW and RW-R were only observed for 16S rRNA (0.0451), *bla_CTX-M32_* (0.0096), and *sul1* (0.0115). Therefore, the chlorination treatment was not efficient in the removal or degradation of ARGs. These results are in agreement with the literature, where several authors have reported that a high dose of chlorine is necessary to achieve a significant reduction in ARGs.^15^ For example, Zang demonstrated that low concentrations of free chlorine (5, 10, 15, and 20 mg/L) removed <0.4 Log of *sul1, intI1*, and 16S rRNA, while a higher concentration (30 mg/L) could achieve 1.2–1.3 Log removal.^57^ In addition, Yuan reported a 0.4 and 0.1 Log reduction of erythromycin and tetracycline resistance genes at 15 mg Cl_2_ min/L in wastewater.^58^

The ARG removal may be related to the oxidative mechanism of chlorination. HOCl first attacks the cell wall (proteins and peptidoglycan) and the cell membrane.^59,60^ Second, it can penetrate and reach the cytoplasm, causing damage to nucleic acids (purine and pyrimidine), the main targets being thymidine, deoxy-guanosine, and uridine monophosphate.^59^ Consequently, it can be assumed that gene removal requires higher chlorine concentrations, but this comes with the undesirable formation of toxic and carcinogenic DBPs, which may increase the chemical risks associated with the reuse of RW.^61^ In addition, it has been described that DBPs, due to their effects on bacteria (reactive oxygen species formation, SOS response activation, increased cell permeability, and altered expression of conjugation-relevant genes), can promote and accelerate ARG dissemination between bacteria, especially by the conjugative transfer mechanism.^62^

In fact, until now, ARG removal remains a major challenge, as very low removal (<1 LRV) by advanced wastewater treatments at full-scale facilities or even an increase in the relative abundance of some ARGs has been reported. Although ozonation has proven to be an efficient treatment for the removal of OMCs at full-scale UWWTPs, a poor efficiency in removing ARGs has been shown. Keenu monitored ARGs in a field water reuse treatment train, which consists of ozone followed by biologically active carbon and granular activated carbon (O_3_/BAC/GAC). Three main conclusions can be pointed out from this study: (i) a low quantitative removal of ARGs (0–1 LRV); (ii) enrichment of the total ARG relative abundance (respect to 16S rRNA), and (iii) reduction in the number of “clinically relevant” ARGs.^63^ Moreover, the application of full-scale ozonation leads to no measurable effect on intracellular ARGs (specifically, *sul1*).^64^ On the other side, UV-C-based AOPs, which represent another treatment type with high prospects to be widely implemented at full scale, have also been shown to have a negligible efficiency: ca. 0.4 and 0.2 LRV for UV-C alone (42 J/L) and UV-C/oxidant (0.5 mM of peroxymonosulfate or H_2_O_2_), respectively.^65^ Therefore, the impact of the UWWTP processes on ARGs is a complex problem. In fact, the results obtained at full-scale indicates that another strategy, different from the one for disinfection and OMC removal, should be implemented to alleviate ARG pressure in the environment. In this context, to increase the removal of ARGs, the secondary treatment stage can be a potential tool to improve the global capability of ARG removal in UWWTPs, maybe through mixed separative technologies, such as membrane bioreactor (MBR), which has been shown to have a high efficiency on ARG removal (from 1.5 to 7.3 LRV).^66^

#### 3.4.2 Greenhouses

The ARGs (log units per 100 mL) detected in the secondary reservoirs and drip systems of the four greenhouses are shown in Table 2. In general, most of the monitored ARGs were present in all samples and at concentrations close to or similar to those of RW-R, with some exceptions where significant increases or decreases in percentages and statistical differences were observed (Table S9).

**Table 2:** Quantification of ARGs in Secondary Reservoirs (R) and Dropped Water (D) of the Four Greenhouses^a^

		G1	G2	G3	G4
		Log units/100 mL ± SD			
16S rRNA	R	8.87 ± 0.23	9.82 ± 0.33	10.00 ± 0.18	9.26 ± 0.64
	D	9.39 ± 0.23	9.81 ± 0.17	9.10 ± 0.98	9.47 ± 0.93
*intI1*	R	6.40 ± 1.13	8.65 ± 0.99	8.58 ± 1.17	6.25 ± 0.76
	D	8.34 ± 0.10	8.65 ± 0.17	7.36 ± 1.83	7.23 ± 2.48
*bla_CTX-M32_*	R	<LOQ	6.54 ± 0.58	7.10 ± 1.23	6.53 ± 0.58
	D	7.42 ± 1.08	6.56 ± 0.47	7.09 ± 0.82	7.08 ± 1.00
*sul1*	R	3.34 ± 1.10	6.32 ± 0.19	7.25 ± 0.06	5.23 ± 0.94
	D	6.25 ± 0.51	6.43 ± 0.78	5.69 ± 1.00	5.41 ± 1.79
*bla_TEM_*	R	<LOQ	5.58 ± 0.38	6.78 ± 0.44	5.31 ± 0.51
	D	6.20 ± 1.05	5.49 ± 0.12	5.99 ± 0.79	6.29 ± 0.77
*qnrS*	R	<LOQ	<LOQ	6.32 ± 0.54	<LOQ
	D	5.91 ± 0.46	5.03 ± 0.56	5.12 ± 1.15	5.24 ± 0.53

a LOQ (Log units/100 mL): 16S rRNA (4.7), *intI1* (3.6), *Bla_CTX-M32_* (6.0), *Sul1* (3.0), *Bla_TEM_* (4.87), and *qnrS* (4.9).

In R1 (filled with 100% groundwater), only 16S rRNA, *intI1*, and *sul1* were detected at low concentration; therefore, statistical differences (*p*-value <0.05) regarding RW-R, R2, R3, and R4 were observed (Table S8). The occurrence of these genes in natural water (surface water, groundwater, well water) has already been reported,^67^ including sulfonamide, aminoglycoside, β-lactamase, tetracycline, and chloramphenicol resistance genes. In contrast, and due to the direct use of RW-R for irrigation, all genes were detected in D1 at slightly lower levels (<10% difference in concentration) compared to RW-R.

In the case of R2, the levels of 16S rRNA and *intI1* were similar to those of RW-R, while ARGs experienced a 10–25% reduction. ARG concentrations detected in the corresponding drip system (D2) did not show any reservoir-related variation, except for *qnrS*, where an unexpectedly high concentration (ca. 18%) was detected.

Gene concentrations in R3 were very similar to those in RW-R, except for *qnrS* that showed a 10% increase. In contrast, D3 showed the largest reduction in gene concentrations (10–20%) compared to the reservoir.

Finally, the values detected in R4 (75% groundwater) were significantly lower than those in RW-R (7–28%) for all genes, similar to R1. However, in contrast to the previous greenhouses, all gene concentrations increased in the drip irrigation system, especially for *intI1* (15.7%), *bla*_*TEM*_ (18.5%), and *qnrS* (11.3%).

The observed differences between the ARG concentrations measured in reservoirs and drips of the evaluated greenhouses were likely due to the design of the RW distribution system. The few articles addressing this issue^52,68^ attributed these differences to bacterial regrowth and/or biofilm proliferation inside the tubing and pipes of the system.^6^ Fahrenfeld detected ARGs (*sul1* and *tetA*) at the end of the RW distribution system, even at higher concentrations.^6^ Nevertheless, other authors investigated the potential spread of ARGs at the end of the RW distribution system and found no differences in ARG concentrations between the point of entry and the point of use for *sul1*, *qnrA*, and *bla_TEM_*.^52^

The tertiary treatment system evaluated at real scale, based on sand filtration and chlorination, showed an adequate performance and complied with the current EU water reuse legislation regarding microbiological quality of class A (as the concentration of *E. coli* detected was <10 CFU/100 mL). However, the use of secondary water reservoirs and long storage periods increased the water microbiological load, confirming the direct use of RW as a safer practice.

Regarding the occurrence of naturally occurring ABs, the chlorination treatment removed only 58% of the initial load of antibiotics. Unlike the microbiological load, water storage contributes to a slight reduction of the AB concentration in irrigation water, probably due to photolytic degradation.

On the other hand, as expected, chlorination is not efficient in the removal of ARGs, highlighting as well the risk of spreading new genes into the environment. The presence of *intl1*, a horizontal gene transfer responsible for the AR spreading, and all of the ARGs has been detected in all stages of the reclaimed water, as well as reservoirs and dropper’s samples.

The results obtained in the present study carried out in a real-scale water treatment scenario revealed the inefficacy of the chlorination to remove and control emerging contaminants, such as ABs, ARB, and ARGs, reinforcing the need for applying effective treatments and water distribution strategies (such as the direct reuse immediately after the treatment) to minimize the potential environment and human risks associated with water reuse in agriculture, and also supporting the actions included into the new Directive EU 2024/3019 regarding the regulation of OMCs and, in a near future, ARGs.

## Supplementary Material



## Notes

Additional contents for quantification of antibiotics by LC-MS/MS, DNA extraction and PCR amplification procedure by qPCR; additional tables for data of target antibiotics, MS/MS transitions and LOQs, ARG standard curves, physicochemical parameters in treatment plant and greenhouses (reservoirs and droppers), average levels of antibiotics and bacteria in reservoirs and droppers of the greenhouses, and ARG ratios of reservoirs and irrigation droppers (PDF)
