# Supplementary material for: From the Reclaimed Water Treatment Plant to Irrigation in Intensive Agriculture Farms: Assessment of the Fate of Antibiotics, Antibiotic Resistance Bacteria and Genes, and Microbial Pathogens at Real Scale
Source: Environ Sci Technol. 2025 Sep 9;59(37):19953–65. doi: 10.1021/acs.est.5c02823 (PMC12461941; doi:10.1021/acs.est.5c02823)

Title: From the Reclaimed Water Treatment Plant to Irrigation in Intensive Agriculture Farms: Assessment of the Fate of Antibiotics, Antibiotic Resistance Bacteria and Genes, and Microbial Pathogens at Real Scale.

Authors: Flor X. Cadena-Aponte[1,2], Patricia Plaza-Bolaños[1,2], Ana Agüera[1,2*], Samira Nahim-Granados[1,3], Ilaria Berruti[1,3], María Jesús Abeledo-Lameiro[1,3], Maria Inmaculada Polo-López [1,3*]

Affiliations:

[1]Solar Energy Research Centre (CIESOL), Joint Centre of the University of Almería-CIEMAT, Carretera de Sacramento s/n, Almería, 04120, Spain

[2]Department of Chemistry and Physics, University of Almería, Carretera de Sacramento s/n, Almería, 04120, Spain

[3]Plataforma Solar de Almería-CIEMAT, Carretera de Senés Km 4, 04200, Tabernas, Almería, Spain.

SUPPLEMENTARY MATERIAL

***CORRESPONDING AUTHORS:**

Dr. María Inmaculada Polo-López

Plataforma Solar de Almería-CIEMAT, Carretera de Senés Km 4, 04200, Tabernas, Almería, Spain. Phone: + 0034 950 387900-987

E-mail: mpolo@psa.es

Prof. Ana Agüera

Department of Chemistry and Physics, University of Almería, Carretera de Sacramento s/n, Almería, 04120, Spain Phone: + 0034 950 015531

E-mail: aaguera@ual.es

This document provides more detailed information about the main paper mentioned above. The following information is included:

Contents	Description	Page
**Content S1**	Antibiotics (ABs) quantification by LC-MS/MS.	S3
**Content S2**	PCR amplification procedure by qPCR.	S4
**Table S1**	List of target antibiotics, MS/MS transitions and estimated limits of quantification (LOQs).	S5
**Table S2**	Equations of the standard curve of all ARGs.	S7
**Table S3**	Physicochemical parameters of UWW along the reclamation plant and statistical analysis.	S8
**Table S4**	Main physicochemical parameters of UWW in the greenhouses (R: reservoir and D: drop water) and statistical analysis.	S9
**Table S5**	Ionic contents of UWW in the greenhouses (R: reservoir and D: drop water).	S10
**Table S6**	Average levels of antibiotics detected in the reservoirs (R1-R4) of the greenhouses (concentrations are shown in ng/L).	S11
**Table S7**	Average levels of antibiotics detected in the irrigation droppers (D1-D4) of the greenhouses (concentrations are shown in ng/L).	S12
**Table S8.**	Microbial concentration (CFU/100 mL) detected along UWW reclamation plant and subsequent secondary reservoirs and droppers' water of the four greenhouses, and ANOVA analysis.	S13
**Table S9**	ARGs statistical analysis (Bonferroni test/ANOVA) and ratio of increment and reduction percentage of reservoirs (R) and droppers (D) of the four greenhouses.	S14
**Figure S1**	Sampling points diagram.	S15

Content S1. Antibiotics (ABs) quantification by LC-MS/MS.

High purity AB standards (>95%) were purchased from Sigma-Aldrich (Darmstadt, Germany), Dr. Ehrenstorfer GmbH (Augsburg, Germany), Fluka (Steinheim, Germany), HPC (Cunnersdorf, Germany) and TRC Canada (Toronto, Canada). The selected ABs were determined in the water samples by liquid chromatography coupled to quadrupole-lineal-ion-trap mass spectrometry (LC-QqLIT-MS/MS). An Exion AC chromatograph (Sciex, Foster City, CA, USA) was coupled to a 5500 QTRAP™ (Sciex) mass spectrometer. LC-MS solvents were supplied by Honeywell (Hannover, Germany). A Pursuit PFP analytical column (150 x 2.1 mm, 3 µm, Agilent, Middelburg, The Netherlands) was used for the chromatographic separation and the mobile phases consisted of water (0.1% formic acid) (A) and methanol (B). The gradient was as follows: 10% B (1 min), 100% B in 8 min (hold 4 min), and 10% B in 0.5 min (hold 3 min). The total analysis time was 15.5 min, the injection volume was 20 µL and the mobile phase flow was 0.4 mL/min. The column oven and autosampler temperature were set at 30ºC and 15ºC, respectively. The direct injection technique was applied, and the only sample pre-treatment was the filtration of the samples using PTFE syringe filters (0.22 µm). Injection vials were prepared at a proportion of water:MeOH (90:10, *v/v*) containing ^{13}C-caffeine as internal standard (1000 ng/L). Samples were diluted (1:10 or 1:100, *v/v*) with water (keeping the composition water:MeOH, 90:10, *v/v*) to quantify saturated compounds when necessary.

The electrospray source (Turbo IonSpray) was operated in the positive mode (ESI+) for all the compounds with the following settings: ion spray voltage (IS), 5500 V; source temperature, 500 ºC; CAD gas, medium; ion source gas 1 (GS1), 50 psi; ion source gas 2 (GS2), 40 psi and curtain gas, 9 (arbitrary units). Nitrogen was used as a nebuliser, curtain and collision gas. The multiple reaction monitoring (MRM) mode and the Scheduled MRM™ algorithm were used to acquire the samples. An MRM detection window of 40 seconds was applied, and the target scan time was 1 second. The following criteria confirmed the analytes: two SRM transitions with the expected retention time (tolerance ± 0.1 min) and an appropriate SRM2/SRM1 ion ratio (tolerance ±30%). The target ABs were quantified by preparing matrix-matched calibration curves to minimise the matrix effect. The estimated limits of quantification (LOQ) and MS parameters can be found in **Table S1**. Analyst™ and Sciex OS™ were used for data acquisition and data processing, respectively.

Content S2. PCR amplification procedure by qPCR.

Quantification was performed in duplicate for each sample using a standard curve specifically performed in this study for the selected genes and following the same methodology described elsewhere (*Arslan-Alaton, I.; Karatas, A.; Pehlivan, Ö.; Koba Ucun, O.; Ölmez-Hancı, T., 2021. Effect of UV-A-assisted iron-based and UV-C-driven oxidation processes on organic matter and antibiotic resistance removal in tertiary treated urban wastewater. Catalysis Today 361, 152–158).*

The standard curves (for all ARGs) were performed by using the plasmid Seq_01/21ABUTPC (Invitrogen, Thermo Scientific). This plasmid is a synthetic sequence containing the fragments of the ARGs. The standard curves were constructed using serially 10-fold dilutions of the stock received from the manufacturer to obtain standards ranging from $1{\cdot}10^3$ to $1{\cdot}10^{11}$ copies/µL, calculated from the concentration of the genes contained in the plasmid, according to equation (Eq 1):

$$DNA\left(\frac{copies}{\mu L}\right) = \frac{6.023\times10^{23}\left(\frac{copy}{mol}\right)\times DNA\ amount\left(\frac{g}{\mu L}\right)}{DNA\ lenght(bp)\times 660\left(\frac{\frac{g}{mol}}{bp}\right)} \qquad \text{(Eq. 1)}$$

The C_t (threshold cycle) values for each dilution were measured in duplicate and plotted against the logarithm of their DNA copy number. The standard curve of all ARGs studied was generated by linear regression of the plotted points. Therefore, to quantify the copy number of ARGs in each water sample, Eq (2) was used according to the standard curve data (**Table S2**) (*Lee, C., Kim, J., Shin, S.G., Hwang, S., 2006. Absolute and relative QPCR quantification of plasmid copy number in Escherichia coli. Journal of Biotechnology 123, 273-280)*).

$$\frac{Copies}{100\ mL} = \frac{10^{\frac{(C_t-a)}{b}}\times D_f}{V} \qquad \text{(Eq. 2)}$$

where C_t is the value to the intersection between an amplification curve and a threshold line obtained by qPCR, *a* is the constant value obtained from the standard curve (y = a + bx), *b* is the slope value obtained from the standard curve, D_f is the dilution rate of the total DNA sample used as a template for qPCR, and *V* is the volume of the extracted sample.

Table S1. List of target antibiotics, MS/MS transitions and estimated limits of quantification (LOQs).

Compound	Class	LOQ[a] (ng/L)	RT[b] (min)	Precursor ion (*m/z*)	DP[c] (V)	Product ion (*m/z*)	EP[d] (V)	CE[e] (V)	CXP[f] (V)
Amoxicilin	β-Lactam	500	3.73	366.1	70	114	12	30	19
						208	12	13	8
						349.1	6	18	11
Azithromycin	Macrolide	10	7.08	749.5	50	83.1	11	110	20
						591.4	12	40	13
						573.3	8	47	14
Cephalexin	β-Lactam	100	4.83	348	60	158.1	12	13	8
						174.1	11	20	11
					150	106.1	10	47	11
Cefotaxime	β-Lactam	100	5.11	456.1	40	324.1	5	15	3
						396.1	5	10	2
						241.3	5	20	2
Chlortetracycline	Tetracycline	100	6.15	479.2	100	444	9	31	10
						462	9	24	11
Ciprofloxacin	Fluoroquinolone	50	6.38	332.2	50	314.3	5	25	2
						231.2	5	48	2
Clarithromycin	Macrolide	10	7.97	748.4	45	158.4	5	35	2
						590.4	5	26	2
Clindamycin	Lincosamide	10	6.49	425.2	80	126.1	11	35	7
						377.1	12	28	9
Danofloxacin	Fluoroquinolone	50	6.26	358.2	100	340.2	8	31	12
						314.3	8	26	11
Difloxacin	Fluoroquinolone	10	6.78	400.3	70	299	8	42	17
						356	8	30	20
Doxycycline	Tetracycline	50	6.78	445.3	90	428.2	10	28	9
						410.2	10	34	10
						154.1	10	39	10
Enrofloxacin	Fluoroquinolone	10	6.42	360.3	80	245.2	10	37	10
						316.2	10	27	12
Erithromicyn	Macrolide	10	7.41	734.6	58	158.3	5	40	2
						576.5	5	28	2
Flumequine	Quinolone	10	7.35	262.3	50	244.08	5	21	14
						202.3	5	41	12
Josamycin	Macrolide	10	8.04	828.6	54	174.2	12	46	13
						229.1	11	43	11
						600.2	11	37	14
Levofloxacin + Ofloxacin	Fluoroquinolone	10	6.06	362.1	80	261.2	8	40	10
						318.2	8	26	15
Lincomycin	Lincosamide	10	4.60	407.1	50	126.3	5	45	2
						359.3	5	23	2
Metronidazole	Nitroimidazole	10	3.64	172.1	35	128.1	5	20	2
						82.1	5	30	2
Nalidixic acid	Quinolone	10	6.89	233.2	45	187	10	35	12
						104	10	55	11
Norfloxacin	Fluoroquinolone	100	6.23	320	220	302.2	12	33	17
						233.1	12	33	5
Oxytetracycline	Tetracycline	50	5.52	461.3	90	426.1	10	27	11
						443.1	10	19	11

Roxithromycin	Macrolide	10	7.80	837.5	140	158	8	43	15
						679.4	8	31	10
Sulfadiazine	Sulfonamide	10	4.11	251.2	40	92.1	5	35	2
						10.1	5	30	2
Sulfamethazine	Sulfonamide	10	4.85	279	42	186.2	5	20	2
						156.1	5	26	2
Sulfamethizole	Sulfonamide	10	5.13	271.1	80	156.1	6	20	8
						92	6	37	10
						108.1	6	34	10
Sulfamethoxazole	Sulfonamide	10	5.48	254.2	47	156.1	5	21	2
						108.1	5	30	2
Sulfapyridine	Sulfonamide	10	4.37	250.1	47	156.1	5	21	2
						108.3	5	34	2
Sulfathiazole	Sulfonamide	10	4.36	256.2	45	156	5	18	2
						92.2	5	34	2
Tetracycline	Tetracycline	50	5.61	445.2	75	154.2	10	40	11
						410.2	10	27	9
Trimethopim	Diaminopyrimidine	10	5.01	291.3	45	230.2	5	28	2
						123.2	5	30	2
^{13}C-Caffeine	Internal standard		5.10	198.1	40	140.1	5	30	2
						112.2	5	35	2

Abbreviations: [a]LOQ, limit of quantification; [b]RT, Retention time; [c]DP, Declustering potential; [d]EP, Entrance potential; [e]CE, Collision energy; [f] CXP: Collision cell exit potential.

Table S2. Equations of the standard curve of all ARGs.

Gene	Amplicon size (bp)	Standard curve	R^2	LOQ (copies/µL)	Efficiency (%)
**16S rRNA**	195	y=45.63544 - 3.55337x	0.996	1000	90
intI1	196	y=40.70134 -3.01101x	0.995	100	114
sul1	162	y=38.51748 -3.09367x	0.998	10	110
qnrS	118	y=45.34640 - 3.36773x	0.996	850	98
$\boldsymbol{bla_{TEM}}$	113	y=47.43483 - 3.76866x	0.995	750	84
$\boldsymbol{bla_{CTX\text{-}M32}}$	156	y=53.65800 - 4.47996x	0.999	10000	67

Abbreviations: bp: base pairs, LOQ: limit of quantification

Table S3. Physicochemical parameters of UWW along the reclamation plant and statistical analysis.

	**UWW (A)**	**RW-F (B)**	**RW-Ch (C)**	**RW-R (D)**	**ANOVA[a]**	**Bonferroni test [b]**
**Parameters**	av ± SD	av ± SD	av ± SD	av ± SD	p-value	p-value
pH	7.6 ± 0.3	7.5 ± 0.3	7.4 ± 0.3	7.6 ± 0.2	0.190	-
Turbidity (NTU)	7.4 ± 1.8	5.6 ± 2.1	4.3 ± 1.2	6.4 ± 3.0	0.010*	A-C (0.003)
Conductivity (mS/cm)	2.9 ± 0.2	2.9 ± 0.2	3.0 ± 0.2	3.0 ± 0.3	0.009*	C-D (0.038)
HCO_3^- (mg/L)	486.8 ± 66.1	490.2 ± 66.6	489.3 ± 67.8	537.9 ± 83.6	0.002*	A-D, B-D C-D (<0.0001)
DOC (mg/L)	17.6 ± 2.8	17.5 ± 3.8	17.6 ± 3.1	19.2 ± 4.4	0.999	-
Ionic content (mg/L)						
Cl^-	497.5±32.8	487.9 ± 29.9	498.6 ± 46.5	489.7 ± 81.0	0.292	-
NO_2^-	1.8±1.0	1.0 ± 0.6	0.98 ± 0.9	4.8 ± 7.3	0.0001*	A-B (0.038) B-D (0.045)
NO_3^-	2.7±0.9	2.9 ± 1.0	2.8 ± 0.7	2.7 ± 1.2	0.504	-
PO_4^{3-}	6.8±2.4	6.5 ± 2.8	7.03 ± 2.5	7.2 ± 3.6	0.295	-
SO_4^{2-}	123.0±7.0	120.3 ± 8.0	118.9 ± 11.5	124.9 ± 23.4	0.238	-
Na^+	278.1±13.6	271.6 ± 11.3	283.7 ± 16.5	290.7 ± 23.2	0.113	-
NH_4^+	69.2±16.4	67.8 ± 14.2	66.5 ± 13.8	74.9 ± 16.4	0.0001*	A-C (0.038) A-D, B-D, C-D (<0.0001)
K^+	31.3±4.1	30.9 ± 4.0	31.2 ± 4.1	28.2 ± 6.1	0.014*	A-D (0.003) C-D (0.006)
Mg^{2+}	51.5±5.6	51.3 ± 4.9	48.2 ± 10.2	50.7 ± 6.0	0.305	-
Ca^{2+}	95.6±15.1	96.6 ± 11.3	96.8 ± 13.0	90.3 ± 21.1	0.710	-

av ± SD (averaged value ± standard deviation) DOC: Dissolved Organic Carbon.
[a]ANOVA p-value estimated at the significant level 0.05 and referred to the same parameter along the entire treatment system.
[b]Bonferroni test from ANOVA estimated at the significant level of 0.05 and referred to the specific differences between comparison of the different treatment steps.
* P-values < 0.05 indicate significant differences among means of data.

Table S4. Main physicochemical parameters of UWW in the greenhouses (R: reservoir and D: drop water) and statistical analysis.

	RW-R	R1	D1	R2	D2	R3	D3	R4	D4	Bonferroni test P-value
**pH**	7.6±0.1	8.0±0.6	6.6±0.5	7.9±0.5	6.4±0.5	7.4±0.2	6.5±0.6	8.1±0.1	7.3±0.5	RW-R-R3 (0.0019), RW-R-R4 (0.0302)<br>R1-R3 (0.00132), R1-D1 (<0.0001)<br>R2-R3 (0.03787), R2 D2 (<0.0001)<br>R3-R4 (<0.0001), R3-D3 (<0.0001)<br>R4-D4 (<0.0001)
**Turbidity (NTU)**	6.0±2.8	1.4±1.0	7.5±3.6	12.2±4.7	21.1±11.8	8.9±6.4	14.8±7.62	3.8±2.2	5.4±2.0	RW-R-R1 (0.0413)<br>R1-R2 (<0.0001), R1-R3 (0.0043)<br>R1-D1 (<0.0001)<br>R2-R3 (0.0101), R2-R4 (<0.0001)
**Conductivity (mS/cm)**	3.0±0.3	4.8±0.5	4.5±0.5	2.8±0.2	3.6±0.7	2.6±0.4	4.4±2.0	4.2±0.3	5.5±1.6	RW-R-R1 (<0.0001)<br>R1-R2 <0.0001), R1-R3 <0.0001)<br>R2-D2 (0.0163)<br>R3-D3 (0.0028)
**HCO_3^- (mEq/L))**	9.3±1.1	5.3±0.8	6.6±2.6	6.4±0.9	4.2±1.4	7.3±1.3	4.7±2.5	7.5±1.1	6.9±1.8	RW-R-R1 (<0.0001), RW-R-R2 (<0.0001), RW-R-R3 (0.0114)<br>R1-R3 (<0.0001), R1-R4 (<0.0001)<br>R2-D2 (<0.0001)<br>R3-D3 (0.0018)
**DOC (mg/L)**	19.5±4.8	6.7±3.0	17.7±2.2	17.8±2.5	20.6±3.6	14.0±2.3	18.3±5.3	4.5±1.6	20.6±11.4	RW-R-R1 (<0.0001), RW-R-R4 (0.0010)<br>R1-R2 (<0.0001), R1-R3 (<0.0001)<br>R1-D1 (<0.0001)<br>R2-R4 (<0.0001)<br>R3-R4 (<0.0001)<br>R4-D4 (0.00111)

Bonferroni test from ANOVA refers to the comparison between the RW-R and R and the corresponding R and D. In the table are included those comparisons with significant differences among means of data, i.e., p-values < 0.05. The pairs of data with no significant differences (p-value > 0.05) are not included.

Table S5. Ionic contents of UWW in the greenhouses (R: reservoir and D: drop water).

Ions (mg/L)	RW-R	R1	D1	R2	D2	R3	D3	R4	D4
Cl^-	484.8±90.9	649.5±86.8	506.0±91.4	528.5±40.7	519.6±42.2	492.±53.6	505.6±53.0	568.2±53.1	610.9±77.8
NO_2^-	1.6±0.8	1.1±0.3	12.6±16.9	12.3±7.2	10.1±8.3	3.7±1.4	5.9±4.5	1.2±0.7	1.2±0.6
NO_3^-	2.7±1.4	5.4±2.8	614.5±295.5	2.9±1.4	410.4±268.5	6.9±5.9	588.5±221.6	15.2±4.8	624.9±281.7
PO_4^{3-}	7.0±3.4	-	71.1±51.0	5.8±1.7	119.8±84.1	6.2±1.7	55.1±38.5	1.6±0.4	166.7±209.5
SO_4^{2-}	124.4±26.5	997.0±142.3	184.5±104.9	137.5±13.2	134.5±25.2	126.0±12.9	132.1±14.2	900.7±160.9	1052.7±251.5
Na^+	293.5±25.3	628.0±91.7	260.0±88.4	294.0±23.0	286.3±28.5	274.0±26.9	272.6±30.6	522.8±32.1	524.1±28.9
NH_4^+	80.0±13.3	1.2±0.9	144.1±57.0	45.0±13.4	43.7±13.3	57.3±14.7	72.3±21.4	3.5±2.7	134.8±116.1
K^+	28.6±3.2	45.7±8.2	205.3±71.7	29.2±2.4	166.9±111.4	26.9±3.6	136.3±80.8	36.2±8.91	303.0±185.2
Mg^{2+}	50.2±6.7	103.3±16.9	50.6±4.8	49.9±4.3	48.2±8.1	46.6±6.3	48.1±6.7	94.3±16.5	91.3±22.5
Ca^{2+}	91.9±15.9	141.5±25.2	158.6±56.8	98.8±13.3	128.5±56.5	92.9±11.8	172.9±55.9	178.4±26.8	208.5±69.6

Table S6. Average levels of ABs detected in the reservoirs (R1-R4) of the greenhouses (concentrations are shown in ng/L).

	Greenhouse 1 (R1)				***Greenhouse 2 (R2)***				***Greenhouse 3 (R3)***				***Greenhouse 4 (R4)***			
	**ATL[a]**	**SD[b]**	**FD (%)[c]**	**Min-Max[d]**	**ATL**	**SD**	**FD (%)**	**Min-Max**	**ATL**	**SD**	**FD (%)**	**Min-Max**	**ATL**	**SD**	**FD (%)**	**Min-Max**
**∑ Antibiotics**	**44**	**56**	**-**	**26-178**	**1548**	**1128**	**-**	**462-4452**	**1809**	**1584**	**-**	**338-5964**	**88**	**86**	**-**	**10-272**
Azithromycin	21	10	20	19-22	364	459	100	27-1440	498	580	100	32-2015	47	83	80	10-262
Clarithromycin	n.d.	-	-	-	13	6	20	10-15	12	7	70	10-18	10	5	20	10-10
Erithromycin	n.d.	-	-	-	44	31	80	15-97	67	38	80	43-141	10	5	30	10-11
Levofloxacin[e]	62	50	60	21-159	561	322	100	243-1262	646	641	100	57-2291	44	33	50	12-83
Metronidazole	n.d.	-	-	-	18	10	70	10-30	45	38	90	11-106	10	-	10	10
Sulfadiazine	n.d.	-	-	-	12	6	30	10-14	13	7	40	10-17	n.d.	-	-	-
Sulfamethoxazole	n.d.	-	-	-	447	354	100	45-1398	374	269	100	110-1071	29	17	50	15-45
Sulfapyridine	n.d.	-	-	-	35	19	90	14-54	95	42	100	29-150	n.d.	-	-	-
Trimethoprim	14	-	10	14	91	41	100	30-155	88	37	100	39-158	26	15	30	11-36

[a] ATL: average total load (n = 10). [b] SD: Standard deviation. [c] FD: Frequency of detection. [d] Concentration range showing the minimum and maximum concentration. [e] Sum of levofloxacin and the enantiomer ofloxacin. n.d.: not detected.

Table S7. Average levels of antibiotics detected in the irrigation droppers (D1-D4) of the greenhouses (concentrations are shown in ng/L).

	Greenhouse 1 (D1)				*Greenhouse 2 (D2)*				*Greenhouse 3 (D3)*				*Greenhouse 4 (D4)*			
	**ATL[a]**	**SD[b]**	**FD (%)[c]**	**Min-Max[d]**	**ATL**	**SD**	**FD (%)**	**Min-Max**	**ATL**	**SD**	**FD (%)**	**Min-Max**	**ATL**	**SD**	**FD (%)**	**Min-Max**
**∑ Antibiotics**	**2705**	**1336**	**-**	**1498-5734**	**1485**	**732**		**733-3007**	**1935**	**1419**		**653-5761**	**107**	**62**		**34-224**
Azithromycin	1016	592	100	266-1921	403	386	100	90-1311	556	551	100	40-1883	39	30	70	10-96
Clarithromycin	23	11	78	11-43	14	7	18	12-16	12	5	73	8-15	n.d.	-	-	-
Erithromycin	67	35	100	15-131	35	22	100	12-83	57	35	82	6-138	n.d.	-	-	-
Levofloxacin[e]	939	568	100	314-2111	535	324	100	123-1189	740	540	100	257-2057	62	45	70	21-128
Metronidazole	48	24	100	29-103	16	8	72	10-35	42	35	100	12-111	n.d.	-	-	-
Sulfadiazine	28	16	22	24-32	14	8	18	10-20	14	9	36	1-23	n.d.	-	-	-
Sulfamethoxazole	422	289	100	173-1148	363	155	100	233-783	384	307	91	100-1179	22	12	40	18-29
Sulfapyridine	97	39	100	39-145	37	17	100	10-58	102	55	91	30-180	n.d.	-	-	-
Trimethoprim	104	34	89	3-162	94	31	100	40-131	94	36	100	37-164	n.d.	-	-	-

[a] ATL: average total load (n = 9 (D1); n = 11 (D2, D3), n = 10 (D4). [b] SD: Standard deviation. [c] FD: Frequency of detection. [d] Concentration range showing the minimum and maximum concentration. [e] Sum of levofloxacin and the enantiomer ofloxacin. n.d.: not detected.

Table S8. Microbial concentration (CFU/100 mL) detected along UWW reclamation plant and subsequent secondary reservoirs and droppers' water of the four greenhouses, and ANOVA analysis.

	E. coli	**AR-*E. coli***	***Pseudomonas* spp.**	**AR-*Pseudomonas* spp.**
	Median±MAD	Median±MAD	Median±MAD	Median±MAD
**UWW**	335000±241500	4250±2850	665000±495000	500±200
**RW-F**	340000±303500	8800±5500	620000±504500	500±150
**RW-Ch**	7±6	2±1	1±0	1±0
**RW-R**	9±7.5	1±0	1±0	1±0
ANOVA (P-value)	RW-F-RW-Ch (0.0405)	RW-F- RW-R (0.0315)	RW-F-RW-Ch (0.0454)	RW-F-RW-Ch (<0.0001)
**R1**	2.5±1.5	1±0	45.5±36	1±0
**D1**	5±4	2±1	159.5±158.5	1±0
**R2**	300±299	1±0	360000±335600	1±0
**D2**	128±126.5	4±3	577500±549000	255±196
**R3**	600±592	4±3	85200±80400	7±4
**D3**	1200±1179	3±2	80000±75000	21±16
**R4**	200±198	1±0	490±318	1±0
**D4**	500±499	1±0	6400±5000	11±10
ANOVA (P-value)	-	-	D2-D1 (0.00286) D2-D4 (0.00241)	D2-R2, D2-D1, D2-D3, D2-D4 (<0.0001)

MAD: Median absolute deviation. In the table are included those comparisons with significant differences among means of data, i.e., p-values < 0.05. The pairs of data with no significant differences (p-value > 0.05) are not included.

Table S9. ARGs statistical analysis (Bonferroni test/ANOVA) and ratio of increment and reduction percentage of reservoirs (R) and droppers (D) of the four greenhouses.

		G1	G2	G3	G4
**16S rRNA**					
**R/RW-R**	Ratio	-11.2	-1.7	0.3	-7.3
	P-value	<0.0001*	0.0661	1	0.2523
**D/R**	Ratio	-6.0	-0.1	-9.2	2.2
	P-value	0.0049*	0.7852	0,2032	0.2726
intI1					
**R/RW-R**	Ratio	-27.2	-1.6	-2.4	-28.9
	P-value	0.0117*	1	1	<0.0001*
**D/R**	Ratio	-5.20	-0.1	-14.2	15.7
	P-value	0.0340*	1	1	<0.0001*
$bla_{CTX-M32}$					
**R/RW-R**	Ratio	-19.9	-13.2	-5.8	-13.3
	P-value	<0.0001*	0.0025*	1	0.0137*
**D/R**	Ratio	-1.6	0.4	-0.1	8.4
	P-value	<0.0001*	0.3976	1	0.0112*
sul1					
**R/RW-R**	Ratio	-52.7	-10.6	2.5	-26.0
	P-value	<0.0001*	0.0093*	1	0.1783
**D/R**	Ratio	-11.7	1.6	-21.6	3.4
	P-value	<0.0001*	0.9748	0.0324*	0.9299
bla_{TEM}					
**R/RW-R**	Ratio	-28.4	-18.0	-0.4	-22.0
	P-value	0.0587	0.2818	1	0.0236*
**D/R**	Ratio	-8.9	-1.7	-11.7	18.5
	P-value	0.0393*	1	1	0.0239*
qnrS					
**R/RW-R**	Ratio	-13.9	-25.8	10.5	-17.7
	P-value	0.5627	0.0147*	1	0.1778
**D/R**	Ratio	3.4	18.7	-18.9	11.3
	P-value	0.2944	0.1230	0.8052	0.4701

Abbreviations: Ratio D/R, Dropper/Reservoir; Ratio R/RW-R, Reservoir/Reclaimed water in main reservoir. Positive and negative values mean % of increase and reduction, respectively.
*Samples showing statistical differences of the means according to Bonferroni test ANOVA (for values with p-value < 0.05).

Figure S1. Sampling points diagram.

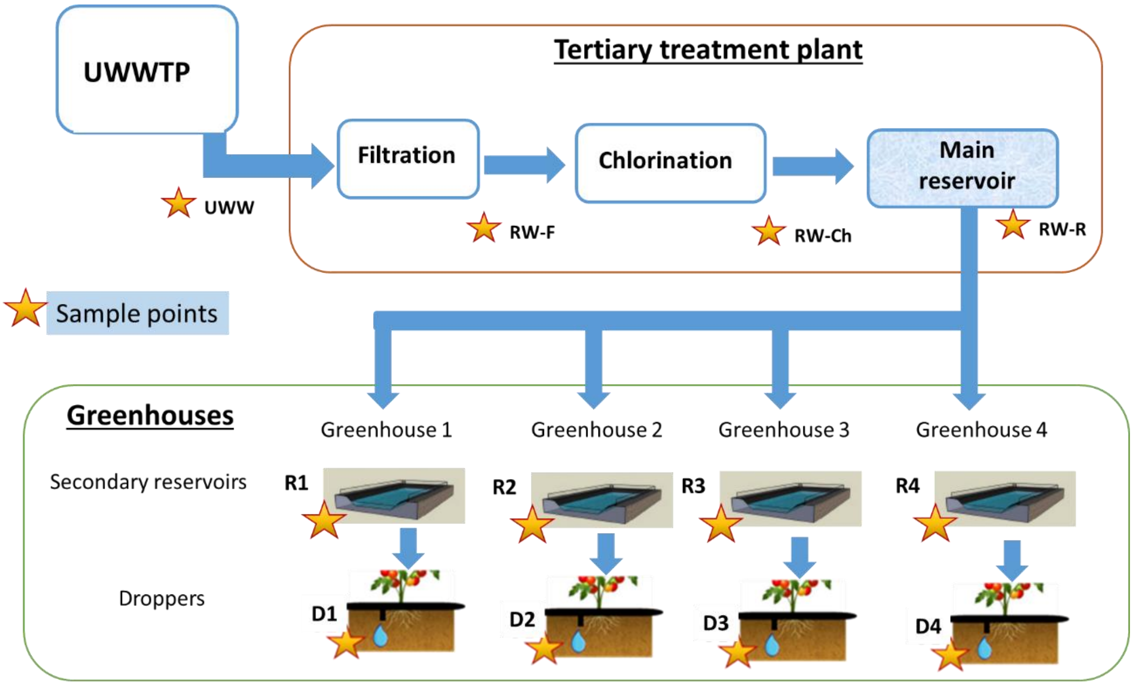

Supplement: Supplementary file 1 [file es5c02823_si_001.pdf]
